# Genome- and Community-Level Interaction Insights into Carbon Utilization and Element Cycling Functions of *Hydrothermarchaeota* in Hydrothermal Sediment

**DOI:** 10.1128/mSystems.00795-19

**Published:** 2020-01-07

**Authors:** Zhichao Zhou, Yang Liu, Wei Xu, Jie Pan, Zhu-Hua Luo, Meng Li

**Affiliations:** a Institute for Advanced Study, Shenzhen University, Shenzhen, People’s Republic of China; b Department of Bacteriology, University of Wisconsin—Madison, Madison, Wisconsin, USA; c Key Laboratory of Marine Biogenetic Resources, Third Institute of Oceanography, Ministry of Natural Resources, Xiamen, People’s Republic of China; Woods Hole Oceanographic Institution

**Keywords:** *Hydrothermarchaeota*, hydrothermal sediment, comparative genomics, carbon utilization, element cycling, community-level interaction, lateral gene transfer

## Abstract

Hydrothermal vents release reduced compounds and small organic carbon compounds into the surrounding seawater, providing essential substrates for microbial growth and bioenergy transformations. Despite the wide distribution of the marine benthic group E archaea (referred to as *Hydrothermarchaeota*) in the hydrothermal environment, little is known about their genomic repertoires and biogeochemical significance. Here, we studied four highly complete (>80%) metagenome-assembled genomes (MAGs) from a black smoker chimney and the surrounding sulfur-rich sediments on the South Atlantic Mid-Ocean Ridge and publicly available data sets (the Integrated Microbial Genomes system of the U.S. Department of Energy-Joint Genome Institute and NCBI SRA data sets). Genomic analysis suggested a wide carbon metabolic diversity of *Hydrothermarchaeota* members, including the utilization of proteins, lactate, and acetate; the anaerobic degradation of aromatics; the oxidation of C_1_ compounds (CO, formate, and formaldehyde); the utilization of methyl compounds; CO_2_ incorporation by the tetrahydromethanopterin-based Wood-Ljungdahl pathway; and participation in the type III ribulose-1,5-bisphosphate carboxylase/oxygenase-based Calvin-Benson-Bassham cycle. These microbes also potentially oxidize sulfur, arsenic, and hydrogen and engage in anaerobic respiration based on sulfate reduction and denitrification. Among the 140 MAGs reconstructed from the black smoker chimney microbial community (including *Hydrothermarchaeota* MAGs), community-level metabolic predictions suggested a redundancy of carbon utilization and element cycling functions and interactive syntrophic and sequential utilization of substrates. These processes might make various carbon and energy sources widely accessible to the microorganisms. Further, the analysis suggested that *Hydrothermarchaeota* members contained important functional components obtained from the community via lateral gene transfer, becoming a distinctive clade. This might serve as a niche-adaptive strategy for metabolizing heavy metals, C_1_ compounds, and reduced sulfur compounds. Collectively, the analysis provides comprehensive metabolic insights into the *Hydrothermarchaeota*.

**IMPORTANCE** This study provides comprehensive metabolic insights into the *Hydrothermarchaeota* from comparative genomics, evolution, and community-level perspectives. Members of the *Hydrothermarchaeota* synergistically participate in a wide range of carbon-utilizing and element cycling processes with other microorganisms in the community. We expand the current understanding of community interactions within the hydrothermal sediment and chimney, suggesting that microbial interactions based on sequential substrate metabolism are essential to nutrient and element cycling.

## Abstract (precis)

This study provides comprehensive metabolic insights into the *Hydrothermarchaeota* from comparative genomics, evolution, and community-level perspectives. Members of the *Hydrothermarchaeota* synergistically participate in a wide range of carbon-utilizing and element cycling processes with other microorganisms in the community. We expand the current understanding of community interactions within the hydrothermal sediment and chimney, suggesting that microbial interactions based on sequential substrate metabolism are essential to nutrient and element cycling.

## INTRODUCTION

Hydrothermal alteration and venting processes transfer reduced sulfur compounds, organic compounds (e.g., C_1_ compounds, petroleum compounds, organic acids), and heavy metals to the surrounding hydrothermal sediments (1–6). Together with deposited sedimentary carbon compounds, these substrates make hydrothermal sediments a distinct ecological niche containing a variety of organic and inorganic nutrients. In hydrothermally active Guaymas Basin sediments, microorganisms syntrophically degrade hydrocarbons and lipids. Indeed, metabolic linkages among microbial groups, such as a substrate-level interdependency between fermentative members and sulfur- and nitrogen-cycling members, were proposed (6). However, the diversity and function of microorganisms that inhabit the hydrothermal environment, especially archaea, remain elusive, and the community-level microbial interactions within this environment have not yet been characterized in detail.

“*Candidatus* Hydrothermarchaeota,” originally identified on the continental slope and abyssal sediments by 16S rRNA gene profiling and termed marine benthic group E (MBG-E) (7), has recently been proposed as a new archaeal phylum (8). According to a previous study, *Hydrothermarchaeota* is abundant in the deep-sea hydrothermal environment, such as the crustal fluids of the Juan de Fuca Ridge (JdFR) flank (9). A combined analysis of metagenome-assembled genomes (MAGs) and single-cell-amplified genomes of *Hydrothermarchaeota* from the crustal fluids of the Juan de Fuca Ridge flank was used to evaluate the evolutionary placement and functional potential of this new archaeal phylum (8). The results of that analysis were further consistent with those of a more recent study (10), with both studies suggesting the potential of *Hydrothermarchaeota* for carboxydotrophy and sulfate and nitrate reduction (8, 10). However, the relatively small number of genomes available to date limits the ability to understand the ecological roles and metabolism of this novel archaeal lineage.

Here, we analyzed the metagenomes from sulfur-rich hydrothermal sediments at an active deep-sea (water depth, 2,770 m) hydrothermal vent site (black smoker chimney layers and the surrounding sulfur-rich sediments) in the southern Mid-Atlantic Ridge of the South Atlantic Ocean (total S content = approximately 100 to 450 mg/g; detailed sample information is provided in Text S1 in the supplemental material). We generated two metagenomic libraries from the layer (the TVG10 library) and surrounding sediments (the TVG13 library) of an active black smoker chimney in the Mid-Atlantic Ridge (BSmoChi-MAR) of the South Atlantic Ocean (38.1 Gb for TVG10 and 30.3 Gb for TVG13). *De novo* metagenome assembly and binning resulted in 140 MAGs (>50% genome completeness) from 24 microbial groups (Table S1), including 5 archaeal MAGs and 135 bacterial MAGs. The metabolic prediction from the resolved MAGs revealed the functional redundancy and syntrophic substrate-utilizing interactions among the microorganisms. Further, based on the four *Hydrothermarchaeota* genomes of relatively high completeness (>80%) obtained in the current study, the results of a previous publication (9), and data in publicly available data sets (Table S2), we propose a metabolic scheme for this archaeal lineage. Finally, the evolutionary analysis suggested the important role of lateral gene transfer (LGT) in the niche adaptation of *Hydrothermarchaeota* to the local environment. The current study provides an in-depth insight into the genomics, community-level interactions, and evolution of *Hydrothermarchaeota*.

10.1128/mSystems.00795-19.1 TEXT S1 Detailed genomic parameters of the assembled genomes. Download Text S1, PDF file, 0.1 MB. Copyright © 2020 Zhou et al. 2020 Zhou et al. This content is distributed under the terms of the Creative Commons Attribution 4.0 International license.

10.1128/mSystems.00795-19.3 TABLE S1 Genomic properties of MAGs resolved from hydrothermal sediment metagenomes. Download Table S1, XLSX file, 0.04 MB. Copyright © 2020 Zhou et al. 2020 Zhou et al. This content is distributed under the terms of the Creative Commons Attribution 4.0 International license.

10.1128/mSystems.00795-19.4 TABLE S2 NCBI SRA information for metagenomes retrieved from the hydrothermal vent sediments and spring sediments. Download Table S2, XLSX file, 0.02 MB. Copyright © 2020 Zhou et al. 2020 Zhou et al. This content is distributed under the terms of the Creative Commons Attribution 4.0 International license.

## RESULTS AND DISCUSSION

### *Hydrothermarchaeota* is a novel archaeal phylum.

**FIG 1:**
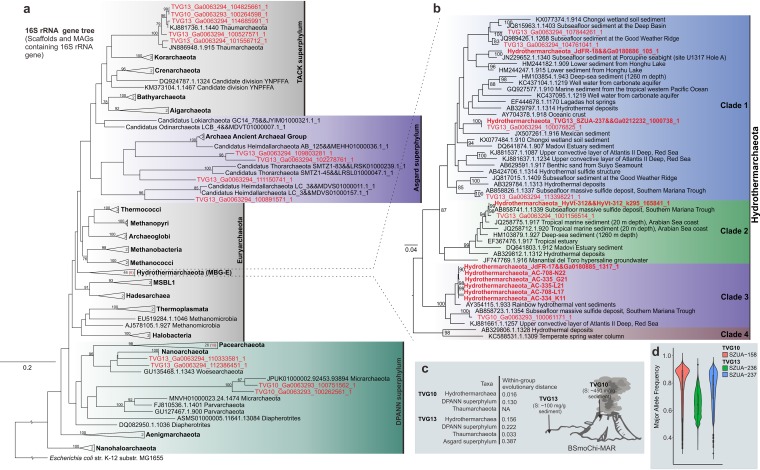
Phylogenetic tree of the 16S rRNA genes from TVG metagenomes. (a) RAxML maximum likelihood tree, constructed by including the metagenome 16S rRNA gene sequences (from both binned and unbinned scaffolds). The sequences obtained in the current study are colored in red; the number in red in parentheses indicates the sequence number from the current study. Bootstrap values over 75% are labeled. The tree was rooted by the Escherichia coli K-12 16S rRNA gene. (b) Detailed *Hydrothermarchaeota* 16S rRNA gene tree. Four clades of the representative sequences of the *Hydrothermarchaeota* 16S rRNA gene were established based on a 90% similarity cutoff. Sequences retrieved from reconstructed MAGs are highlighted in red. (c) Schematic depicting the two sampling locations and the within-group evolutionary distances of the 16S rRNA gene sequences from two samples (calculated by using the Jukes-Cantor model and pairwise comparisons based on global alignment). NA, not available because only one sequence was present within a group and a within-group evolutionary distance could not be calculated. (d) Diagram of the major allele frequency representing the diverse level of potential *Hydrothermarchaeota* in the corresponding environments where the *Hydrothermarchaeota* MAGs were identified.

Reconstructed archaeal MAGs and scaffolds containing phylogenetically informative genes, including those for at least three ribosomal proteins (RPs) or 16S rRNA gene fragments, are summarized in Table 1 and in Table S3 in the supplemental material. Both the 16S rRNA and RP gene phylogenies placed *Hydrothermarchaeota* as a distinct lineage parallel to other *Euryarchaeota* clades, including *Thermococci*, *Methanomicrobia*, and *Hadesarchaea* (Fig. 1a and b and Fig. S1a). Within this lineage, the 16S rRNA gene sequences show median sequence identities of 80.8 to 83.9%, which supports phylum-level diversity (Fig. 1b and Text S1). The phylum name *Hydrothermarchaeota* was originally proposed for this archaeon since all the current known genomes were obtained from hydrothermal sediments or fluids (7–10). The 16S rRNA gene sequence data indicated that members of the *Hydrothermarchaeota* are also widely distributed in wetland, estuarine, marine, and hot spring sediments (10) (Fig. 1b). At the community level, TVG13 covered *Hydrothermarchaeota* 16S rRNA genes more diverse than those covered by TVG10 (Fig. 1c). Meanwhile, from the allele frequency analysis, a higher frequency indicates the dominance of the majority allele over minor alleles. The higher mean major allele frequency on the genome level indicates a less diverse *Hydrothermarchaeota* population in TVG10 than in TVG13 (Fig. 1d).

**TABLE 1:** Overview of genomic statistics for archaeal MAGs constructed in the current study and reference and NCBI SRA deposits^*a*^

Characteristic	Value for the following *Hydrothermarchaeota* MAG:						
	SZUA-158 (TVG10)	SZUA-236 (TVG13)	SZUA-237 (TVG13)	JdFR-16	JdFR-17	JdFR-18	HyVt-292
No. of copies of individual markers							
0	9	21	49	99	87	2	30
1	139	128	136	25	59	145	141
2	1	0	3	25	29	2	17
3	0	0	0	0	12	0	0
4	0	0	0	0	1	0	0
5+	0	0	0	0	0	0	0
Completeness (%)	92.53	84.97	65.82	31.93	53.87	98.13	80.43
Contamination (%)	0.93	0.00	1.76	13.55	25.15	1.87	9.06
Strain heterogeneity (%)	0.00	0.00	33.33	84.00	71.83	0.00	35.29
Bin size (bp)	1,749,358	1,270,198	1,228,833	1,353,114	2,178,134	2,062,134	1,426,722
*N*_50_ (bp)	9,590	6,219	3,825	6,267	7,687	149,032	5,600
Mean scaffold length (bp)	7,706.42	5,747.50	3,510.95	5,614.58	6,331.78	93,733.36	4,988.54
No. of scaffolds	227	221	350	241	344	22	286
GC (%)	38.99	47.58	45.76	49.68	49.64	39.14	40.02
GC SD (%)	1.91	1.99	2.89	3.92	4.50	1.54	1.98
Coding density (%)	84.96	91.48	84.96	91.98	91.94	92.12	89.88

a HyVt-292 was reconstructed from the metagenome of deep-sea massive sulfide deposits from the Southern Mariana Trough (NCBI accession number DRR093004), which is hydrothermally inactive. JdFR MAGs were reconstructed from the data for subsurface ﬂuids of the Juan de Fuca Ridge ﬂank.

10.1128/mSystems.00795-19.2 FIG S1 Genome taxonomy and genome function of *Hydrothermarchaeota*. Download FIG S1, PDF file, 0.7 MB. Copyright © 2020 Zhou et al. 2020 Zhou et al. This content is distributed under the terms of the Creative Commons Attribution 4.0 International license.

10.1128/mSystems.00795-19.5 TABLE S3 Genomic overview of archaeal MAGs from the current study and hydrothermarchaeotal MAGs from NCBI SRA deposits other than the ones listed in Table 1. Download Table S3, XLSX file, 0.01 MB. Copyright © 2020 Zhou et al. 2020 Zhou et al. This content is distributed under the terms of the Creative Commons Attribution 4.0 International license.

### Mixotrophic lifestyle and versatile substrate utilization by *Hydrothermarchaeota*.

**FIG 2:**
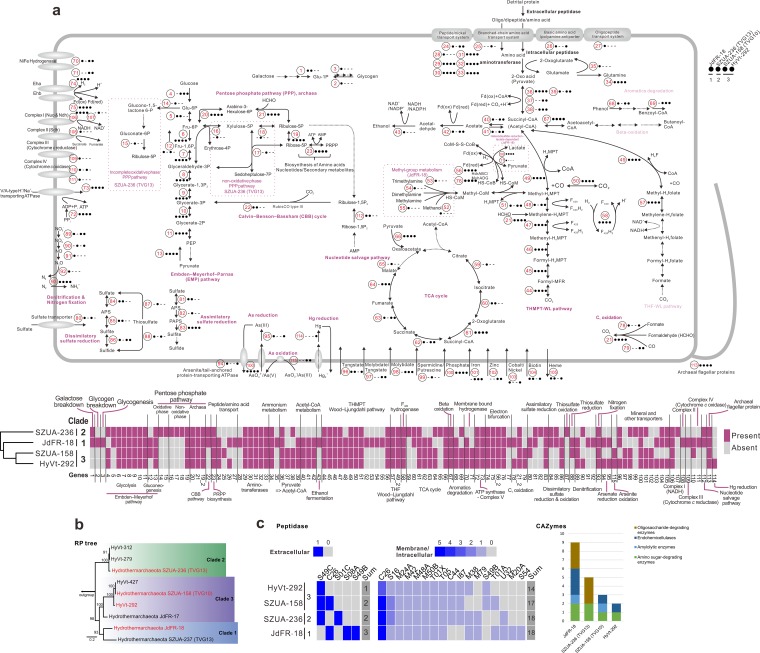
Metabolic pathways in *Hydrothermarchaeota*. (a) Schematic showing the metabolic pathways of the four *Hydrothermarchaeota* MAGs with highly complete genomes. red, reduction; ox, oxidation. (b) RP tree showing the phylogeny of all currently available *Hydrothermarchaeota* MAGs. (c) Heatmap and bar chart depicting the distribution of extracellular/intracellular peptidases and carbohydrate-active enzymes (CAZys) in *Hydrothermarchaeota* MAGs. Information on the glycoside hydrolase (GH) family within each functional category is summarized in the bar chart.

We selected four representative *Hydrothermarchaeota* MAGs (SZUA-158 [TVG10], SZUA-236 [TVG13], JdFR-18, and HyVt-292) with relatively high completeness values (>80%) from the major three clades for metabolic prediction analysis (Table 1, Fig. 2a, Fig. S1b, and Tables S4 and S5). Similar to the findings of a previous study (8), almost all *Hydrothermarchaeota* MAGs contained the tetrahydromethanopterin (THMPT)-based Wood-Ljungdahl (WL) pathway (the THMPT-WL pathway) and some components of the tetrahydrofolate (THF)-based Wood-Ljungdahl pathway (the THF-WL pathway) (Fig. 2a). Since none of these MAGs contained the complete genes for the THF-WL pathway, it might well be that this pathway is not active in *Hydrothermarchaeota* (Fig. 2a). The THMPT-WL pathway in *Hydrothermarchaeota* might function in both directions, either reductively incorporating CO_2_ into acetyl coenzyme A (CoA) synthesis or oxidatively converting products from central carbon metabolism (peptide and sugar carbohydrate degradation) to be channeled into energy-producing pathways. If the former direction is operational, *Hydrothermarchaeota* probably lead a mixotrophic lifestyle, using both inorganic and organic carbon sources. *Hydrothermarchaeota* lacked the methyl coenzyme M reductase (MCR) for methane metabolism. However, JdFR-18 might be able to incorporate a variety of methyl-containing compounds into the WL pathway, including mono-, di-, and trimethylamines and methanol. This feature is frequently observed in members of many other archaeal taxa, such as *Methanosarcinales*, *Methanomassiliicoccales*, *Methanofastidiosa*, *Bathyarchaeota*, and *Verstraetearchaeota* (11). In particular, JdFR-18 (clade 1) encoded HdrD (three copies) and GlcD (four copies; an FAD-containing dehydrogenase and a putative d-lactate dehydrogenase), with one pair of these two genes being colocated and responsible for heterodisulfide reduction linked to lactate utilization. This gene arrangement and lactate utilization have also been observed in the Archaeoglobus fulgidus, *Bathyarchaeota*, and *Verstraetearchaeota* genomes (11–13).

10.1128/mSystems.00795-19.6 TABLE S4 Annotation of four *Hydrothermarchaeota* MAGs. Download Table S4, XLSX file, 0.7 MB. Copyright © 2020 Zhou et al. 2020 Zhou et al. This content is distributed under the terms of the Creative Commons Attribution 4.0 International license.

10.1128/mSystems.00795-19.7 TABLE S5 MEROPS-based peptidases and carbohydrate-active enzymes annotation result. Download Table S5, XLSX file, 0.02 MB. Copyright © 2020 Zhou et al. 2020 Zhou et al. This content is distributed under the terms of the Creative Commons Attribution 4.0 International license.

*Hydrothermarchaeota* genomes probably encode the full tricarboxylic acid (TCA) cycle but not the beta-oxidation pathway (harboring only the acetyl-CoA *C*-acetyltransferase genes; Fig. 2a). Genes encoding benzoyl-CoA reductase subunits (BcrBC) were present in HyVt-292 and JdFR-18, which indicates ATP-dependent benzoyl-CoA degradation. Benzoyl-CoA is the central intermediate of anaerobic degradation pathways of many aromatics, including benzene, phenol, 4-OH-benzoate, cresols, phenylacetate, ethylbenzene, and others (14). Three out of four analyzed *Hydrothermarchaeota* MAGs encoded vanillate/4-hydroxybenzoate decarboxylase subunit C (BsdC) and flavin prenyltransferase (UbiX), which further supports their phenol-degrading activity. The existence of genes for both ADP-forming acetyl-CoA synthetase (Acd; EC 6.2.1.13) and acetyl-CoA synthetase (Acs; EC 6.2.1.1) suggests the possibility of both acetate fermentation and acetogenesis. In addition, further fermentation of acetate to ethanol was evident in some *Hydrothermarchaeota* MAGs, and genes for enzymes from acetaldehyde (by aldehyde ferredoxin oxidoreductase [Aor]) to ethanol (by alcohol dehydrogenase [AdhP or AdhE]) were also identified (Fig. 2a).

### C_1_ oxidation and other element cycling capacities.

*Hydrothermarchaeota* might anaerobically oxidize CO for ferredoxin generation, considering the existence of the gene encoding the carbon monoxide dehydrogenase catalytic subunit (CooS) (clade 3) (8, 15). Both JdFR-18 and HyVt-292 harbored the *fdsABG* operon for NAD^+^-dependent formate dehydrogenase, indicating that *Hydrothermarchaeota* can use formate as a bioenergy source for electron-transferring phosphorylation (16). The fused 3-hexulose-6-phosphate synthase/6-phospho-3-hexuloisomerase (Hps-Phi) (EC 4.1.2.43) and bifunctional enzyme Fae-Hps (EC 4.2.1.147) are responsible for formaldehyde fixation in the ribulose monophosphate cycle (part of the pentose phosphate pathway [PPP]) and the generation of methylene-tetrahydromethanopterin (methylene-H_4_MPT) (the THMPT-WL pathway) (3), which can be further used for the biosynthesis of ribose and acetyl-CoA, respectively. C_1_ compounds of various redox states are common (CO and formate) or potentially available (formaldehyde) and are generated through geochemical reactions in the hydrothermal environments (1–4). Further, some anaerobes generate CO (4). Combined with the CO_2_ fixation ability, which is mainly the Calvin-Benson-Bassham (CBB) cycle and WL pathway, the mixotrophic lifestyle possibly makes *Hydrothermarchaeota* more adaptive within the global benthic environmental setting (17).

In addition, the process of sulfur oxidation to sulfate might take place in *Hydrothermarchaeota* because the dissimilatory sulfite reductase (DsrAB) that they encode can also facilitate sulfur oxidation (8, 18). The existence of key genes for the Sox pathway in SZUA-236 (TVG13) suggests that these organisms have the ability to oxidize thiosulfate for energy yield (Fig. 2a). The potential denitrification and sulfate reduction pathways might enable *Hydrothermarchaeota* to scavenge diverse organic matter by anaerobic respiration. Presumably, *Hydrothermarchaeota* might couple nitrate reduction with the oxidation of reduced sulfur compound (S^0^, S^2–^, and S_2_O_3_^2–^) as an energy-generating process (19). Since the hydrothermal environment is frequently enriched in heavy metals (20), *Hydrothermarchaeota* also possess genomic components for the detoxification of As(V) (arsenate reductase [ArsC] and arsenite/tail-anchored protein-transporting ATPase [ArsA]) and Hg(II) (mercuric reductase [MerA]). They might also oxidize As(III) (cytomembrane-bound arsenite oxidase subunits [AioB]) and presumably couple the reduction of As(V) with the oxidation of reduced sulfur compounds, which suggests that As cycling could constitute one of the energy metabolism pathways in these microorganisms.

### Coexistence of nucleotide salvage pathway and CBB cycle.

The genomes of almost all members of the *Hydrothermarchaeota* encoded the Embden-Meyerhof-Parnas (EMP) pathway for both the glycolysis and gluconeogenesis directions (almost all members encoded fructose-1,6-bisphosphatase [FBP] and phosphoenolpyruvate [PEP] synthase/pyruvate phosphate dikinase [PPDK]) (Fig. 2a). For the former, based on the genome data, the conversion of PEP to pyruvate (catalyzed by pyruvate kinase) was lacking; however, the reverse reactions of PEP synthase/PPDK have been reported in some thermophilic archaea, including *Thermococcus* (*Euryarchaeota*) and *Thermoproteus* (*Crenarchaeota*) (21). All *Hydrothermarchaeota* clades contained an archaeon-style pentose phosphate pathway (PPP) in the genome. In addition, the SZUA-236 (TVG13) genome encoded an incomplete oxidative PPP and a nonoxidative PPP. The PPP and the phosphoribosyl pyrophosphate (PRPP) synthesis pathway are anabolic pathways for the biosynthesis of a variety of amino acids, nucleotides, and other secondary metabolites that utilize substrates from glycolysis (22). They most likely fix CO_2_ by type III ribulose-1,5-bisphosphate carboxylase/oxygenase (RuBisCO) in the CBB cycle, based on genomic predictions (Fig. 2a). The lack of phosphoribulokinase (Prk) of the CBB cycle is frequently reported in archaeal genomes (23). Meanwhile, some reports based on metabolic experiments indicate the presence of autotrophic activity of the crenarchaeotal CBB cycle, despite the lack of Prk. This suggests that although Prk-encoding genes were not detected in the *Hydrothermarchaeota* genomes in the current study, these archaea might nonetheless fix CO_2_ via the CBB cycle (24).

The presence of the nucleotide salvage pathway and CBB cycle suggests that *Hydrothermarchaeota* might recover the RNA/DNA degradation products (such as AMP) for glycolysis or cycle them back into PPP for biosynthesis (Fig. 2a) (25). In addition to RNA/DNA degradation, AMP could also originate from the activities of (i) AMP-forming adenylylsulfate reductase during sulfate reduction (clades 1 and 2), (ii) PRPP synthesis (all clades), and (iii) ADP-dependent (AMP-forming) phosphofructokinase/glucokinase (clade 3) during glycolysis (26–28). The genomic components of type III RuBisCO and the nucleotide salvage function have also been identified in many other euryarchaeotal groups, including *Archaeoglobi*, *Halobacteria*, *Thermococci*, *Hadesarchaea*, and euryarchaeotal methanogens (1). The unconventional participation in the type III RuBisCO-based CBB cycle in the nucleotide salvage function of the above-mentioned archaeal taxa suggests the importance of the primary function of type III RuBisCO in the early stages of archaeal evolution (1, 25).

### Limited carbohydrate assimilation in protein/peptide degraders.

No genes encoding potential sugar and carbohydrate transporters were identified in the three major clades of *Hydrothermarchaeota* in the current study (Fig. 2c). The genomes of these archaea encoded genes with limited functions in carbohydrate assimilation and transformation only for galactose degradation and glycogen conversion. The annotation of carbohydrate-active enzymes (CAZys) also suggested a limited capacity for carbohydrate utilization. Among the peptidases encoded by the *Hydrothermarchaeota* genomes, serine-type endopeptidase S08A is the dominant extracellular peptidase, according to both metagenome and metatranscriptome data, in marine group I (MG-I), -II, and -III archaea from deep-sea hydrothermal plume (29); S49C is an archaeal signal peptide peptidase that destroys cleaved signal peptides; C26 is a gamma-glutamyl hydrolase that probably has glutamine amidotransferase activity (Tables S4 and S5). Further, genomic predictions in the current study indicated that while the genomes of *Hydrothermarchaeota* from clades 1 and 3 encode various peptide/amino acid transporters, the genomes of the three major clades encode the genes of six groups of aminotransferases to transfer amino residues (Tables S4 and S5) and the genes for pyruvate ferredoxin oxidoreductase (Por), indolepyruvate ferredoxin oxidoreductase (Ior), 2-oxoglutarate/2-oxoacid ferredoxin oxidoreductase (Kor), pyruvate dehydrogenase, and dihydrolipoamide dehydrogenase to assimilate 2-oxo acids (pyruvate) to succinyl-CoA (acetyl-CoA) and replenish the energy pool of reducing equivalents. The presence of the various peptide/amino acid transporters and endopeptidases/aminotransferases encoded by the *Hydrothermarchaeota* genomes suggests that they can use detrital peptides/proteins as one of their main carbon and energy sources.

### Functional redundancy and community-level interactions.

**FIG 3:**
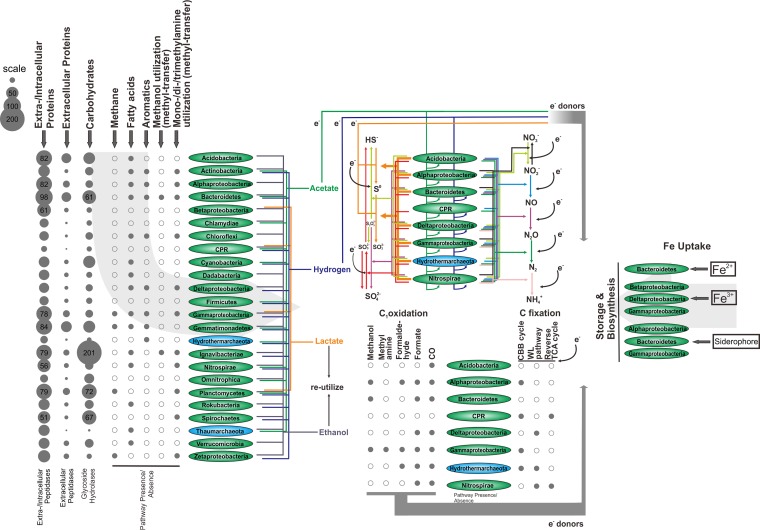
Metabolic prediction for the community of MAGs from TVG metagenomes. The abundance of peptidases and carbohydrate-degrading enzymes was calculated by considering the MAG completeness and taking the average for all MAGs within an individual microbial group (0 digits were used after the decimal point). The presence of a specific pathway/function within each microbial group was assigned when the pathway/function was present in any MAG within this microbial group. The Fe uptake pathway was predicted by use of the previously established database. For the metabolic prediction of N and S cycling, C_1_ oxidation, and C fixation, only the major microbial groups (with at least one MAG from the group having over 15× genome coverage) are represented.

We next analyzed the metabolic capacities of all the reconstructed 140 MAGs (Fig. 3 and Tables S1, S2, and S6) to investigate microbial community interactions. Among these, *Acidobacteria*, *Bacteroidetes*, and *Gemmatimonadetes* possessed the greatest abundance of genes encoding extracellular peptidases. Presumably, they are the major players utilizing detrital proteins in marine sediments. Other microbial groups could engage in syntrophic interactions with the above-mentioned microbes to assimilate extracellular peptides/proteins using the extracellular peptidases secreted by these microbes. Furthermore, *Ignavibacteriae*, *Planctomycetes*, and *Spirochaetes* possessed the most genes encoding glycoside hydrolases, suggesting that they are the major players in carbohydrate/sugar utilization. In addition, a variety of microbial groups are predicted to be able to degrade/utilize methane, fatty acids, aromatics, methanol, and mono-, di-, and trimethylamines. The fermentation products probably include acetate, hydrogen, lactate, and ethanol. The electron pool generated by fermentation is transferred to terminal electron acceptors or CO_2_ for either respiration or carbon fixation. Furthermore, the fermentation products from the first fermentation process could also be reutilized by the community members as energy and carbon sources.

10.1128/mSystems.00795-19.8 TABLE S6 Community metabolic analysis annotation result and KEGG result for the *Euryarchaeota* and *Hydrothermarchaeota* groups. Download Table S6, XLSX file, 0.05 MB. Copyright © 2020 Zhou et al. 2020 Zhou et al. This content is distributed under the terms of the Creative Commons Attribution 4.0 International license.

The eight major microbial groups (with at least one MAG from each group with over 15× genome coverage, including *Acidobacteria*, *Alphaproteobacteria*, *Bacteroidetes*, candidate phyla radiation [CPR], *Deltaproteobacteria*, *Gammaproteobacteria*, *Hydrothermarchaeota*, and *Nitrospira*) are predicted to possess multiple functions for sulfur cycling, including sulfide oxidation, sulfur oxidation, thiosulfate oxidation, sulfate reduction, and thiosulfate disproportionation. The oxidized sulfur compounds (SO_4_^2–^, SO_3_^2–^, and S_2_O_3_^2–^), as well as nitrate/nitrite and molecular oxygen (except for CPR and *Hydrothermarchaeota*), could serve as the terminal electron acceptors for respiration involving organic or inorganic energy sources (Table S6a). This suggests that microorganisms in the chimney layers and surrounding sediments of BSmoChi-MAR have evolved various strategies to adapt to the microaerobic to anoxic environment. In addition to *Hydrothermarchaeota*, several other microbial groups are predicted to oxidize multiple C_1_ compounds and have a carbon fixation capacity, as predicted by the genome contents. Further, some of these microbial groups can probably degrade carbohydrates and peptides/proteins and have sulfur cycling and denitrification abilities, such as the *Alphaproteobacteria*, *Deltaproteobacteria*, *Gammaproteobacteria*, and *Nitrospirae* (Fig. 3 and Table S6a). The inferred redundancy of carbon utilization and element cycling functions among microorganisms and the interactive processes of syntrophic and sequential utilization of substrates might make a wide range of substrates and energy sources available for the community.

### Comparative genomics.

**FIG 4:**
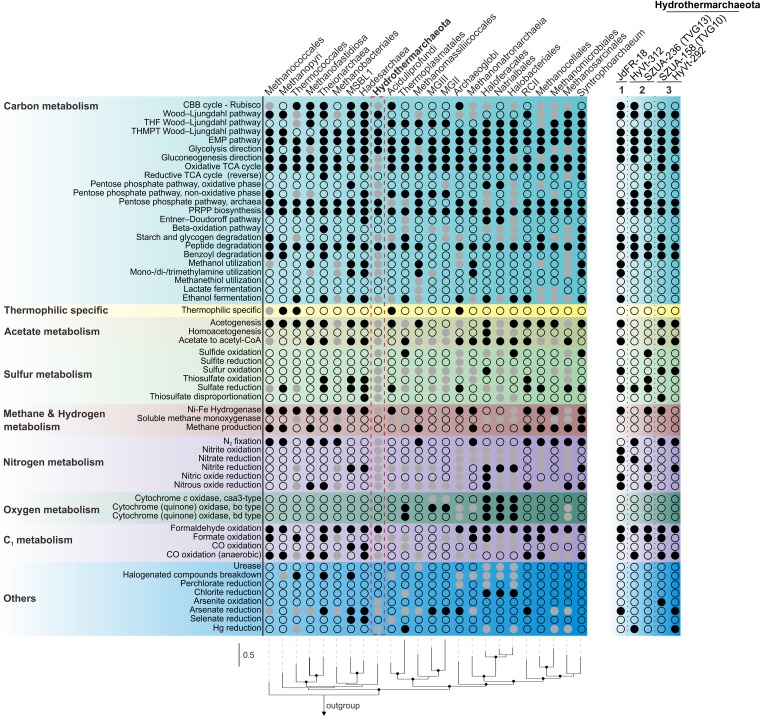
Comparison of the metabolic capacity of *Hydrothermarchaeota* and *Euryarchaeota*. Metabolic marker genes from the Pfam, TIGRfam, and KEGG databases were used to identify the corresponding sequences in up to five genomes within one archaeal group. Solid black dots, solid gray dots, and blank dots, present in all genomes, present in some genomes, and not present in any genome, respectively. If one marker gene was identified, it was assumed that the corresponding metabolic function exists. Because of the limited number of genomes and low genome completeness, solid black dots were used to denote gene identification in one genome for *Hadesarchaea*, *Hydrothermarchaeota*, *Theionarchaea*, *Syntrophoarchaeum*, and MSBL-1. A detailed summary of the information is provided in Text S1 in the supplemental material. The metabolic capacity of five *Hydrothermarchaeota* MAGs is also depicted. The RP phylogenetic tree was constructed by randomly selecting one genome from each group; bootstrap values over 75% are depicted as black dots on the node.

We next selected representative genomes from the euryarchaeotal groups and *Hydrothermarchaeota* to compare their metabolic capacities. While a peptide degradation capacity was shared by most *Hydrothermarchaeota* and euryarchaeotal groups, other carbohydrate-degrading or -utilizing capacities, such as for starch/glycogen, aromatics, fatty acids, methanol, and mono-, di-, and trimethylamines, were distributed in a patchy manner (Fig. 4, Fig. S1c, and Table S6b). In comparison with the euryarchaeotal groups, the *Hydrothermarchaeota* genomes encoded multiple components for the cycling of N and S and the *Hydrothermarchaeota* could oxidize three important C_1_ compounds (formate, formaldehyde, and carbon monoxide). At the within-phylum level, a distinct distribution of metabolic traits among the three major clades within the *Hydrothermarchaeota* was apparent (Fig. 4). Unlike other clades, clade 1 MAG (from the subsurface ﬂuids of JdFR [SubFlu-JdFR]) might specifically utilize methanol, methanethiol, and mono-, di-, and trimethylamines. Although the chemical profile of the Juan de Fuca Ridge flank subsurface fluids is lacking (9), genomic evidence suggested the existence of methyl compounds in the surrounding environment. Clade 3 MAGs (SZUA-158 from the chimney layer of BSmoChi-MAR and HyVt-292 from sulfide deposits of the Southern Mariana Trough) were capable of sulfur oxidation (DsrAB) as the major sulfur-cycling function, which is probably attributed to the high levels of sulfide in the chimney layer and sulfide deposits. Presumably, clade 3 also mainly depended on sulfide oxidation for energy generation and exhibited limited sugar carbohydrate degradation (Fig. 2c). Collectively, the comparative genomic analysis indicated that each clade possesses metabolic traits related to niche adaptation.

### Clade-distinctive LGT.

**FIG 5:**
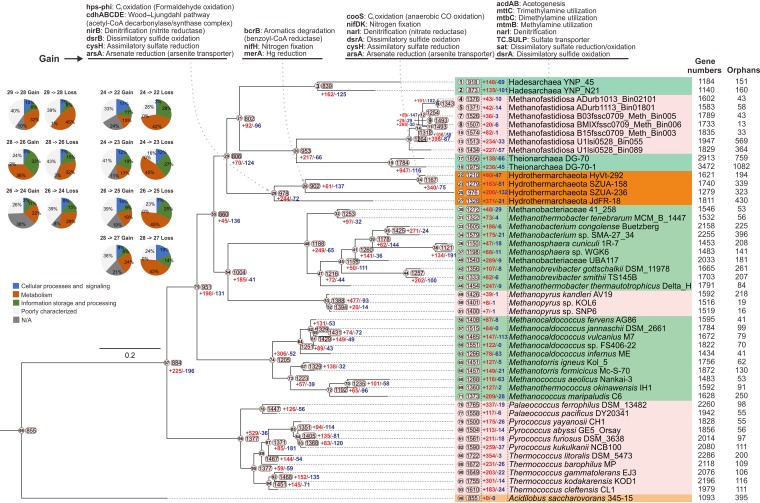
Estimation of orthologous group (OG) turnover events for *Hydrothermarchaeota* and related euryarchaeotal orders and classes. The numbers of OGs (numbers in rectangles) and inferred OG gain and loss numbers (red and blue numbers) are labeled accordingly on the tree nodes and tips. The numbers in circles indicate the order of nodes and tips. The COG category information of the OGs gained or lost for the *Hydrothermarchaeot*a clade was parsed and is depicted. Important genes that were involved in the OG gain events in the *Hydrothermarchaeota* clade were also labeled at the corresponding nodes.

**FIG 6:**
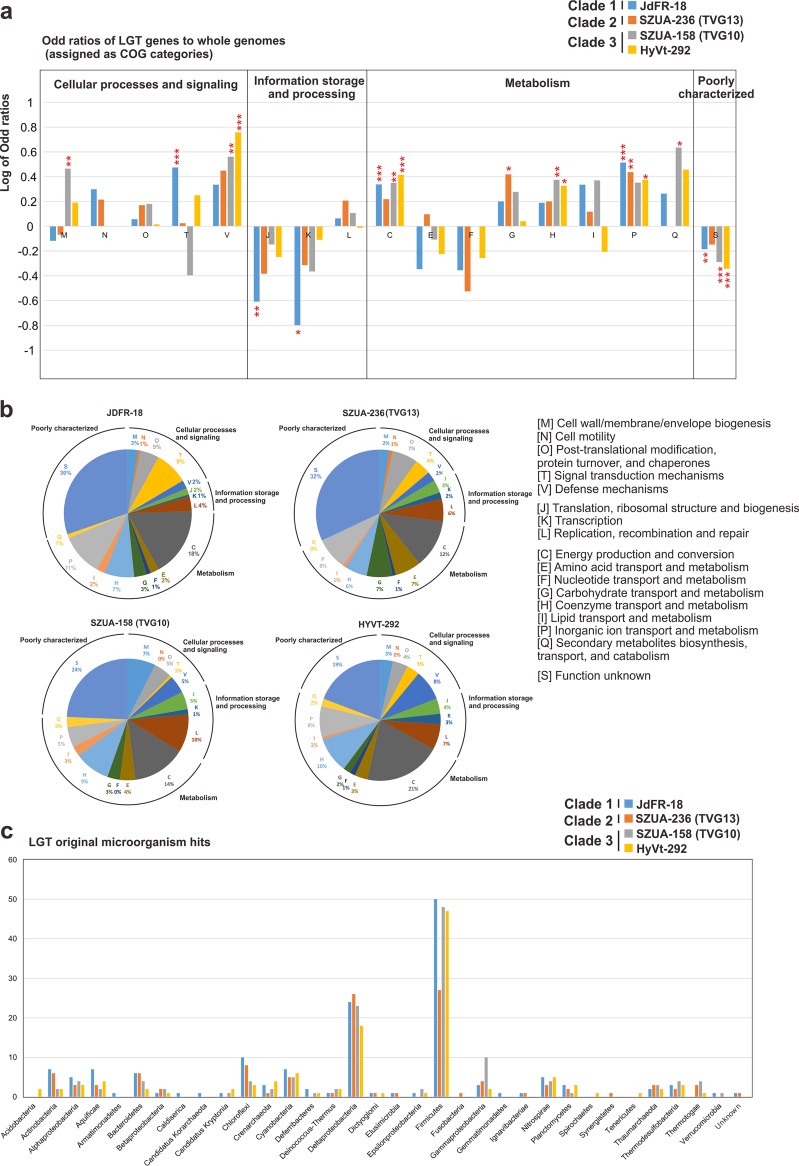
Lateral gene transfer analysis of *Hydrothermarchaeota*. (a) Odds ratios of potential genes horizontally transferred to whole genomes for genes assigned COG categories. The *P* value and odds ratios were calculated by Fisher’s exact test. The *P* values indicate significant deviations between horizontally transferred genes and whole genomes. *, *P* < 0.05; **, *P* < 0.01; ***, *P* < 0.001. (b) Distribution of COG categories of potential genes horizontally transferred. (c) Distribution of the original microorganisms of potential hits or horizontally transferred genes. Microorganisms were divided into the phylum level.

We also mapped the minimum parsimony-based prediction of gene gain and gene loss events of inferred gene orthologous groups (OGs; synonymous with gene families) to the RP-based phylogenomic tree (Fig. 5). Seven phylogenomically closely related euryarchaeotal classes or orders, including methanogens and nonmethanogens, were included. The key gene gain events at nodes 28, 26, and 24 (occupying 24.9%, 6.7%, and 29.1% of the ancestral genomes, respectively) indicated that important traits of extant *Hydrothermarchaeota* have been derived from LGT. These represent many functions, such as C_1_ oxidation of formaldehyde and carbon monoxide, CO_2_ fixation (key components of the WL pathway), and acetyl-CoA synthesis, nitrogen and sulfur cycling, aromatic degradation, and Hg and As reduction. The gene gain events at node 27 (occupying 27.9% of the genome) probably enabled JdFR-18 to utilize tri-, di-, and monomethylamines, also allowing acetogenesis and nitrogen and sulfur cycling (Table S7a). The lost OGs at these nodes have functions mainly associated with amino acid transportation and metabolism, energy production and conversion, and transcription and translation (Fig. 6 and Table S7a). As indicated above, *Hydrothermarchaeota* might have derived functional components via LGT interactions between community members in the hydrothermal environment as an adaptive strategy in an ecosystem containing copious amounts of heavy metals, C_1_ compounds, and reduced sulfur compounds (1–6, 19, 20, 30). The distinctive C, H, N, and S metabolism of each clade (Fig. 4 and 5 and Tables S7b and c) could also be an outcome of LGT events during adaptation in the corresponding econiches. The LGT events could result from various mechanisms, such as symbiosis (31), virus-mediated gene transfer (32), and/or recombination between microorganisms (33). Nevertheless, the LGT-derived functions probably render *Hydrothermarchaeota* a distinctive archaeal lineage able to adapt to the hydrothermal environment.

10.1128/mSystems.00795-19.9 TABLE S7 Lateral gene transfer analysis of *Hydrothermarchaeota*. Download Table S7, XLSX file, 1.0 MB. Copyright © 2020 Zhou et al. 2020 Zhou et al. This content is distributed under the terms of the Creative Commons Attribution 4.0 International license.

### Conclusions.

The phylum *Hydrothermarchaeota* is a widespread archaeal lineage in the hydrothermal sediment environment. Within the microbial community, *Hydrothermarchaeota* synergistically participate in a wide range of carbon utilization and element cycling processes with other microbes. This suggests that microbial interactions are essential for nutrient and element cycling and extends the current understanding of community interactions within hydrothermal sediment and chimney environments (6, 34). Furthermore, the functional redundancy of carbon utilization and element cycling greatly increases the access of *Hydrothermarchaeota* to a wide range of carbon and energy sources.

*Hydrothermarchaeota* members have derived important functional components from the community via LGT. The comparative and evolutionary genomic analysis presented in the current study revealed that *Hydrothermarchaeota* members inhabiting the hydrothermal sediments form a distinctive clade in terms of genome content. LGT appears to have served as a niche-adaptive strategy of individual lineages, enabling them to metabolize heavy metals, C_1_ compounds, and reduced sulfur compounds in the surrounding environment.

Originally detected in but not limited to hydrothermal environments, continental slopes, and abyssal sediments, we suspect that additional uncultured *Hydrothermarchaeota* members dwell in nonextreme environmental settings beyond the hydrothermal ecosystems, such as wetland, estuarine, coastal, and hot spring environments. The findings presented herein call for genomic studies of *Hydrothermarchaeota* from these other environments to further explore the evolutionary mechanisms and strategies used by these archaea to expand and adapt to a variety of environment types. Furthermore, the combined approaches, including genomic, transcriptomic, and enzymatic techniques, in cultivation-based experiments should facilitate research on the metabolic capacities and activities of this fascinating archaeal group in the future.

## MATERIALS AND METHODS

### Sample information and metagenome sequencing.

Marine hydrothermal sediment samples were retrieved from an active deep-sea hydrothermal vent site (black smoker) at a depth of 2,770 m in the Mid-Atlantic Ridge of the South Atlantic Ocean during the DY125-26 cruise of R/V *Dayang Yihao* (R/V *Ocean No. 1*) in August 2012 (35). TVG10 was sampled from a layer from a black smoker chimney; TVG13 is a sulfur-rich sediment sample collected near the black smoker chimney. The samples were collected by the GTV04 deep-sea remote live-video-guided sampling device (maximum diving depth, 4,000 m). The sampler is a remotely operated mechanical clamp which is controlled by a metal shell-armed optoelectric cable to maintain a vertical attitude when sinking to the seafloor. The deep-sea camera system may be adjusted to the best position and attitude to operate the sampling work. Samples were stored at −80°C for subsequent metagenome sequencing, and physicochemical characterizations were conducted soon after collection. The latter characterizations included the total C, H, and S content, C/N ratio, and pH (35) (the chemical experimental details and results refer to those from the previous work). The metagenomic DNA was isolated from the samples using the custom procedure of the FastDNA spin kit for soil (MP Biomedicals, USA), and triple DNA extracts were used and pooled for individual samples.

### Metagenome processing and genome-resolved binning.

To obtain high-quality archaeal MAGs, a custom processing method involving two rounds of assemblage and binning was adopted. The metagenomes were sequenced by using an Illumina HiSeq 2000 platform. Two separate libraries for each sample were obtained and combined into one during downstream analysis. Raw reads were first dereplicated and processed by use of the Sickle tool (https://github.com/najoshi/sickle) with the default settings to trim low-quality reads. Clean reads for each sample were subjected to *de novo* metagenome assembly by use of the IDBA-UD (v1.1.1) assembler with the –mink 52 –maxk 92 –step 8 settings (36). The initial assemblies were deposited in the Integrated Microbial Genomes (IMG) system of the U.S. Department of Energy-Joint Genome Institute (DOE-JGI) database and annotated by using the DOE-JGI Microbial Genome Annotation Pipeline (MGAP; v.4) (37).

Assemblies were subjected to MetaBAT (v0.32.4) tool-based binning with 12 combinations of parameters (38). Subsequently, the Das-Tool (v1.0) program was applied to screen the MetaBAT bins, leading to high-quality and high-completeness bins (39). The CheckM (v1.0.7) program was used to assess the bin quality and phylogeny (40). The subsequently obtained archaeal MAGs were combined with (i) all available archaeal genomes from the GenBank database (updated 2 August 2017); (ii) archaeal clone, fosmid, and cosmid sequences from the NCBI nucleotide database (updated 2 August 2017); and (iii) the initial assembled scaffolds with one or more open reading frames annotated as being of archaeal origin by the IMG database (only assemblies obtained in the current study) as the reference for read mapping. The BBmap program was used to obtain potential archaeal reads from raw reads with the vslow minid = 0.6 option (41). The second round of assembly by archaeal reads was assembled using the same *de novo* metagenome assembling method as that of the first round, and potential archaeon-related scaffolds were submitted to the DOE-JGI IMG database for annotation using the same method as that of the first round. The same method with the MetaBAT and Das-Tool programs was used to generate second-round MAGs; only high-quality archaeal MAGs were used for downstream analysis. Outlier scaffolds with abnormal coverage, tetranucleotide signals, and GC patterns within potentially highly contaminated MAGs (checked by use of the CheckM program) and erroneous small-subunit (SSU) sequences within MAGs were screened and decontaminated by using the RefineM (v0.0.20) program with the default settings (42). Average genome coverages were calculated by remapping raw reads to MAGs using the Bowtie2 (v2.2.8) program (43). Bacterial MAGs were obtained using binning and decontamination processes similar to those described above, but with only one round of binning. Further refinement was also conducted by manual inspection based on the VizBin program for selected MAGs (44).

Information was obtained from the SRA by using the string (((hydrothermal) AND metagenomic[source]) AND WGS[strategy])) NOT 16S[title] NOT 454 GS[text word] AND (metagenome[organism] OR hydrothermal vent metagenome[organism] OR marine sediment metagenome[organism] OR marine metagenome[organism] OR subsurface metagenome[organism]) (updated 26 December 2017) to search hydrothermal vent sediment studies and the string (((spring sediment) AND metagenomic[source]) AND WGS[strategy]) NOT 16S[title] NOT 454 GS[text word] (updated 24 January 2018) to search for freshwater spring sediment studies deposited in the NCBI SRA. The information on the *Hydrothermarchaeota* 16S rRNA distribution and currently available *Hydrothermarchaeota* MAGs and single-cell-amplified genomes suggests that hydrothermal/spring sediments are their most frequently encountered environments. We applied metagenomic data sets from these environments to search for potential *Hydrothermarchaeota* MAGs. The SRA search results were manually inspected to confirm correctness (see Table S1 in the supplemental material). The DOE-JGI IMG deposits linked to these SRA deposits were identified, and the corresponding assemblies were used. MAGs originating from hydrothermal vent sediments (21 studies) and freshwater spring sediments (22 studies) were reconstructed from the public NCBI SRA deposits and the linked DOE-JGI IMG deposits (only one study had the IMG record but no SRA record; that study was also manually inspected to verify that it met the search criteria) (Table S1). SRA runs within one experiment and studies for one biosample underwent integrated assembly. Assembly was conducted by using the MEGAHIT (v1.1.2) program (45) with kmer iterations of k35 to k75, k45 to k95, k65 to k145, and k145 to k295 for 85-bp, 100-bp, 150-bp, and 300-bp reads, respectively, and a kmer step of 10; the preprocessing method was the same as that described above. The assembled metagenomes and reads from studies with DOE-JGI IMG deposits that passed quality control were simply used. The downstream binning methods were the same as those described above, but with one round of binning. Further refinement was also conducted by manual inspection based on the VizBin program for selective MAGs (44).

### Archaeal MAG annotation.

Knockout (KO) annotation was made by use of the GhostKOALA (v2.0), KAAS (v2.1), and eggNOG-mapper (v4.5.1) programs (using the first KO hit and cluster of orthologous groups [COG] hit, COG were translated to KO by use of the ko2cog.xl tool provided by the KEGG database) (46–48). For annotation by use of the NCBI nr database (updated 6 March 2017), the BLASTP result was further refined by extracting only the first meaningful hits (hits with meaningful information rather than a label as a hypothetical protein). Peptidases were called by use of the MEROPS database (using the pepunit database to avoid potential false-positive hits) via the DIAMOND BLASTP (v0.9.10.111) program with the -k 1 -e 1e-10 –subject-cover 80 –id 50 settings (49, 50). The CAZy annotation was done by using the dbCAN (v20170913) program and interpreted by using the CAZy database (self-parsed online information) (51, 52). The InterProScan (v5.26-65.0, client version) software package was used to classify protein functions, with annotations including CDD, PfamA, SMART, TIGRFAM, Phobius, and SuperFamily (53). The Phobius, PRED-SIGNAL, and PSORTb (v3.0.2) (Archaea) programs were used to predict the locations of peptidases as membrane/intracellular or extracellular (only congruent results for the extracellular location from the three methods were adopted; incongruent results were assigned a membrane/intracellular location) (54–56).

### Major allele frequency analysis.

For major allele frequency analysis, the anvi’o (v4.0) program was used to identify and profile single-nucleotide variants (SNVs) of *Hydrothermarchaeota* MAGs based on the mapping of the reads from the corresponding metagenomes. The strategies used to characterize the SNVs identified were performed according to the online instructions (http://merenlab.org/2015/07/20/analyzing-variability/). The major allele frequency value was the percentage of metagenomic reads mapping to a certain site with the majority SNV.

### Comparative genomic analysis.

The Markov cluster (MCL) algorithm implemented in the anvi’o (v4.0) program was used for protein clustering (57). The eggNOG-mapper (v4.5.1) program was used to annotate MAGs with default settings (47, 48). COG functional categories and orthologous groups parsed from the eggNOG mapping results were used to reconstruct the inner tree. The existence of specific functions or pathways was assigned according to the existence of marker genes (using the annotation results obtained as described above). Average nucleotide identity (ANI) values among *Hydrothermarchaeota* MAGs were calculated by use of the OrthoANI program with the default settings (58).

### Phylogenetic reconstruction.

A search of SILVA SSU128 for long marine benthic group E (*Hydrothermarchaeota*) sequences with good quality (pintail quality > 75%, sequence length > 1,000 nucleotides, sequence quality > 75%) resulted in 549 sequences (which were assigned to the *Hydrothermarchaeota* backbone tree HydroBBTree) (59). The alignment obtained was subjected to clustering by use of mothur software (60); 36 representative operational taxonomic unit sequences at a 90% similarity cutoff were obtained. Representative sequences in the SSURef_NR99_128_SILVA database and archaeal 16S rRNA gene sequences retrieved from metagenomic scaffolds (curated by the IMG database) and MAGs were combined (only sequences with lengths of >300 bp were considered) and subsequently aligned by using the SINA (v1.2.11) program (61). The updated 16S rRNA genes from the *Pacearchaeota* and *Asgard* superphylum genomes (deposited in the NCBI Genome database) were also used for tree construction. The SINA alignment obtained with Escherichia coli K-12 as the outgroup was filtered by the use of both ssuref:archaea (LTPs128_SSU) and 50% consensus filters and was subsequently used for tree construction by use of the RAxML-HPC (v8) program on the XSEDE portal implemented in the CIPRES portal with the settings of GTRCAT and 1,000 bootstrap iterations (62, 63).

The 16S rRNA gene sequences (>300 bp) that were identified by a BLAST search in the *Hydrothermarchaeota* MAGs constructed based on NCBI SRAs, previous reports, and the current study were aligned by use of the SINA (v1.2.11) program (61) and inserted into HydroBBTree by using the ARB_PARSIMONY quick-add species method in ARB software (64). Some MAGs lacked 16S rRNA gene sequences, which was not unusual, considering the low MAG completeness. The topology of the generated 16S rRNA gene tree was the same as that of HydroBBTree, and the division of clades also remained unchanged.

The masked alignment of 12 RPs (which were processed by the CheckM program and which included the L2, L3, L4, L5, L14, L16, L18, L22, S3, S8, S17, and S19 RPs) was concatenated and then subjected to tree model selection by use of the ProtTest (v3) program (40, 65). Representative archaeal genomes and reported *Hydrothermarchaeota* MAGs were included in the tree together with MAGs and scaffolds from the current study (66). A preselection was imposed on the concatenated alignment to filter sequences with less than three RPs and less than 25% aligned columns; columns with more than 50% gaps were trimmed. The RAxML-HPC (v8) program on the XSEDE portal implemented in the CIPRES portal was used to generate a phylogenetic tree with the best model as PROTGAMMAILG and 1,000 bootstrap iterations (62, 63). The E. coli K-12 genome was adopted as the outgroup (67).

### Evolutionary analysis.

The genomes of phylogenetically closely related archaeal orders/classes were retrieved from the NCBI Genome database. These included the genomes of the methanogenic organisms *Methanobacteriales*, *Methanococcales*, *Methanofastidiosa*, and *Methanopyri* and the nonmethanogenic organisms *Theionarchaea*, *Hadesarchaea*, and *Thermococcales*. One *Crenarchaeota* genome (Acidilobus saccharovorans strain 345-15) was used as the outgroup. The genome-picking criteria were over 80% completeness and less than 10% genome contamination; the only exceptions were two *Theionarchaea* genomes (the only two genomes available within the class) and one *Hadesarchaea* genome (77.6% completeness; one of two genomes available within the class). The analyzed genomes represented different families or genera, if possible. The phylogenomic tree of the 50 genomes retrieved was constructed by using a concatenated masked alignment of 12 RPs and the same method described above but using the IQ-TREE (v1.6.3) program (68) with the settings -m MFP -mset LG,WAG -mrate E,I,G,I+G -mfreq FU -bb 1000, which let the software precalculate the best-fit model for phylogenetic reconstruction.

The OGs of protein-coding genes shared by 50 genomes were parsed out by using the OrthoFinder (v2.2.6) program (69); orphan genes (genes present in only one genome) were not included in the OGs. The BadiRate program was used to estimate the OG turnover rate using the BDI-FR-CSP model (turnover rates-branch model-estimating procedure, being stringent on estimating turnover rates) (70), with the above-described phylogenomic tree being used as the input tree file. The output gene turnover results were parsed to OG turnover results by a custom Perl script. The OGs were annotated by the eggNOG-mapper (v4.5.1) program; each was assigned the majority annotation result. The key OG turnover events on *Hydrothermarchaeota* nodes were parsed; the related genes with function and pathway annotations were summarized.

### Metabolic capacity prediction and comparison.

The genomes of *Euryarchaeota* and *Hydrothermarchaeota* were retrieved from the NCBI Genome database, and five representative genomes (selected from different families, if possible) from each archaeal group were used (9). Only genomes with over 70% completeness were used. If the number of available genomes in one archaeal group was limited (less than five), all genomes were used, regardless of completeness. Metabolic marker genes were retrieved from a custom metabolic gene database and metabolic pathways annotated in the KEGG database (71, 72). The Pfam, TIGRfam, and custom metabolic gene databases were used to scan the genomes with suggested cutoff settings; KOs were assigned to genomes by using the GhostKOALA (v2.0), KAAS (v2.1), and eggNOG-mapper (v4.5.1) programs with default settings (46–48). For each metabolic marker gene, its presence/absence in the archaeal group was denoted by a solid black dot (present in all groups), solid gray dot (present in some groups), or blank dot (present in none of the groups). For each metabolic function, if one marker gene was identified, the presence of the function was assumed. Because of the limited number of genomes and low genome completeness (less than 70%) for *Hadesarchaea*, *Theionarchaea*, *Syntrophoarchaeum*, and MSBL-1 (Mediterranean Sea Brine Lakes 1), if one metabolic marker gene/metabolic function was identified in any genome within the individual archaeal group, the fact was denoted by a solid black dot.

For the community metabolic analysis of MAGs from both metagenomes, a metabolic capacity prediction method similar to the one described above was used. The abundances of peptidases and CAZys were calculated by considering the MAG completeness and taking the average values for all MAGs within an individual microbial group (0 digits were used after the decimal point). The presence of a specific pathway/function within each microbial group was assigned when the pathway/function was detected in any MAG within the microbial group. The Fe uptake pathway was predicted by the corresponding database (73), using the DIAMOND BLASTP (v0.9.10.111) program with the settings -e 1e-20 –query-cover 80 –id 65 (50).

### Data availability.

Accession numbers for the initial assemblies were as follows: IMG accession number 3300003886 for TVG10 and IMG accession number 3300003885 for TVG13. Accession numbers for the second-round assemblies were as follows: IMG accession number 3300020233 for TVG10 and IMG accession number 3300020236 for TVG13. The MAGs that were resolved in the current study are deposited in NCBI under BioProject accession numbers PRJNA385762 and PRJNA480137. Detailed genomic parameters of the assembled genomes are summarized in Text S1. The NCBI and IMG genome accession numbers for the MAGs that were used in this study are NCBI accession number GCA_003230355.1 for SZUA-236, NCBI accession number GCA_003229275.1 for SZUA-237, NCBI accession number GCA_003229935.1 for SZUA-158, NCBI accession number SRR7786998 for HyVt-292, IMG accession number 2728369317 for JdFR-16, IMG accession number 2728369322 for JdFR-17, and IMG accession number 2728369320 for JdFR-18.

10.1128/mSystems.00795-19.10 DATA SET S1 Metabolic profile of seven *Hydrothermarchaeota* MAGs. Download Data Set S1, XLSX file, 0.8 MB. Copyright © 2020 Zhou et al. 2020 Zhou et al. This content is distributed under the terms of the Creative Commons Attribution 4.0 International license.
