# Correction for Zhou et al., “Genome- and Community-Level Interaction Insights into Carbon Utilization and Element Cycling Functions of *Hydrothermarchaeota* in Hydrothermal Sediment”

**DOI:** 10.1128/msystems.00750-22

**Published:** 2022-08-22

**Authors:** Zhichao Zhou, Yang Liu, Wei Xu, Jie Pan, Zhu-Hua Luo, Meng Li

**Affiliations:** a Institute for Advanced Study, Shenzhen University, Shenzhen, People’s Republic of China; b Department of Bacteriology, University of Wisconsin—Madison, Madison, Wisconsin, USA; c Key Laboratory of Marine Biogenetic Resources, Third Institute of Oceanography, Ministry of Natural Resources, Xiamen, People’s Republic of China

## AUTHOR CORRECTION

Volume 5, no. 1, e00795-19, 2020, https://doi.org/10.1128/mSystems.00795-19. On page 15, “Data availability” should have the following corrections.

The metagenome-assembled genomes (MAGs) that were resolved in the current study have been deposited at NCBI under BioProject accession numbers PRJNA385762 and PRJNA480137. All of the MAGs deposited under PRJNA385762 (*n *= 615) are available. For the MAGs deposited under PRJNA480137, three of the seven original data holders provided approval for the release of their MAGs (*n *= 747); the rest of the MAGs (*n *= 1,148) are no longer available under accession number PRJNA480137, to avoid the duplication of being analyzed and uploaded to NCBI or other databases by the original data holders.

For the MAGs that are no longer available under BioProject accession number PRJNA480137, the original data have been deposited at the Integrated Microbial Genomes & Microbiomes (IMG/M) platform. The IMG identifiers (IDs) and the corresponding owners are provided as follows: 3300005275, 3300005278, 3300005298, 3300007999, 3300008000, 3300008019, 3300009013, 3300009585, and 3300009598 (Brian P. Hedlund); 3300005856 (William P. Inskeep); 3300006767, 3300006799, 3300006808, 3300006809, 3300006865, and 3300006952 (Peter F. Dunfield); and 3300009503 (James G. Elkins).

The samples were recovered from an inactive black smoker chimney instead of an active black smoker chimney as described in the Introduction of the original paper.

Additionally, Fig. 1 is corrected below. The black smoke on the top of hydrothermal vent in Fig. 1c was removed.

**Figure 1:**
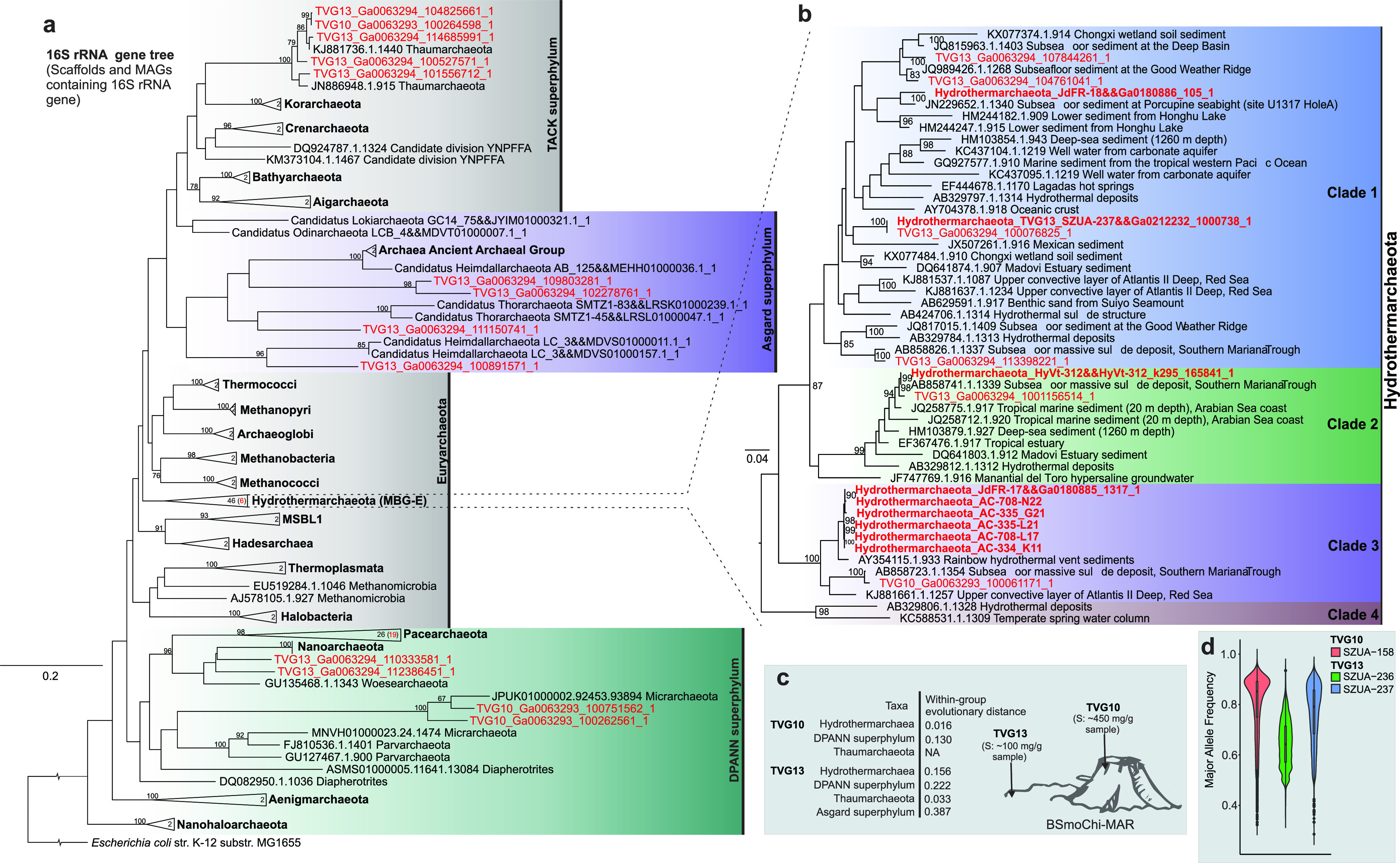


The authors apologize for these corrections. These mistakes and data availability changes do not influence the general conclusions of the paper.

