# Supplementary material for: Genome- and Community-Level Interaction Insights into Carbon Utilization and Element Cycling Functions of Hydrothermarchaeota in Hydrothermal Sediment
Source: mSystems. 2020 Jan 7;5(1):e00795-19. doi: 10.1128/mSystems.00795-19 (PMC6946796; doi:10.1128/mSystems.00795-19)
Supplement: TEXT S1 [file mSystems.00795-19-s0001.pdf]

Supplementary Text S1

The physicochemical parameters of hydrothermal sediment samples

Sample	Depth (m)	pH	Total C (mg/g)	Total N (mg/g)	Total H (mg/g)	Total S (mg/g)	C/N ratio
TVG10N	2770	7.09±0.11	16.81±0.86	2.51±0.61	11.03±1.28	418.81±10.09	7.79±0.76
TVG10W	2770	7.17±0.21	15.47±1.36	2.45±0.04	10.99±0.71	480.73±11.33	6.32±0.65
TVG13	2730	7.30±0.27	19.16±0.91	2.85±0.15	10.95±0.41	104.72±14.303	6.73±0.13

The pH was determined in 1:1 sample/water slurries with an acidometer. The other physicochemical characters were analyzed using a Vario EL III Elemental analyzer (Elementar, Germany). The parameter table and analyzing methods are originated from our previous publication[1]. Sample TVG10N and TVG10W were combined to one, as TVG10; Sample TVG10 and TVG13 were used to isolate metagenomic DNA.

Proposed taxonomic level based on sequence identity range

Taxonomic group	Alternative name	Median sequence identity[a]	Median sequence identity[b]	Proposed taxonomic level based on sequence identity range[c]
Hydrothermarchaeota	Marine Benthic Group E (MBG-E)	83.9	80.8	Phylum

The 16S rRNA gene diversity for assigning taxonomic level was conducted by picking representative sequences by QIIME [2] and being subjected to pairwise sequence identity analysis by BioEdit[3]. The median values of pairwise sequence identities were used.

a. Median sequence identity of representative sequences with 0.97 similarity cutoff from the SILVA database with sequence length over 1400 bp and pintail value over 75 (Nov 22, 2017 updated SSU Ref 128).
b. Median sequence identity of representative sequences with 0.97 similarity cutoff from the SILVA database with sequence length over 1200 bp and pintail value over 75 (Nov 22, 2017 updated SSU Ref 128).
c. This is according to the statistical report from the reference[4].

Archaeal genome reconstruction and phylogeny

According to MAGs and informative scaffolds from both RP and 16S rRNA gene trees, TVG10 acquires more Pacearchaeota and DPANN superphylum communities, while TVG13 acquires more Thaumarchaeota and Asgard superphylum communities, respectively (Figure 1 and Supplementary Figure S1). TVG10 acquires low diversity Hydrothermarchaeota MAGs and scaffolds mainly clustered in Clade 3, while, TVG13 acquires high diversity Hydrothermarchaeota MAGs and scaffolds mainly clustered in Clade 1 and 2 (Figure 1b, 1c). There are minor distributions of Asgard and DPANN superphylum in this regime, albeit, with too low genome coverage for reconstructing MAGs (Figure 1). Comparing to TVG10, TVG13 acquires more diverse Hydrothermarchaeota and DPANN superphylum communities as evident from the analysis on within-group 16S rRNA gene evolutionary distance (Figure 1c). The higher mean major allele frequency of SZUA-158 from TVG10 on the genome level also indicates a less diverse Hydrothermarchaeota population in this environment (Figure 1d). With respect to genomic similarity, Hydrothermarchaeota MAGs discovered in BSmoChi-MAR share low values with MAGs extracted from Juan de Fuca Ridge flank subsurface fluids (SubFlu-JdFR) (Supplementary Figure S2), suggesting the wide phylogenetic distance within this phylum.

Energy conservation metabolism

Components of type 3 and 4 [NiFe] hydrogenases (Hyc and Hyf) are patchily distributed in

Hydrothermarchaeota MAGs; both of these hydrogenases are suggested to function together with formate dehydrogenase for the energy-conserving catalysis of formate to CO_2 and H_2 when the partial pressure of H_2 is low[5]. Energy-converting hydrogenase A (EhaR only) and Energy-converting hydrogenase B (EhbQ only) are also found in all Hydrothermarchaeota MAGs; they are archaeal-specific membrane-associated energy-converting hydrogenases mainly found in hydrogenotrophic methanogens[6]. They catalyze the reversible reduction of ferredoxin by H_2 oxidation which is driven by reverse electron transport[5]. Subsequently, the reduced ferredoxins could serve as electron donor and energy source in the fixation of CO_2 to Formyl-MFR in THMPT-WL pathway[5]. The subsequently oxidized ferredoxins could also serve as electron acceptors in the converting of 2-Oxo acid to acetyl/succinyl-CoA and acetate by a series of ferredoxin oxidoreductase (e.g., Por, Kor, Ior, and Aor); then, the pool of reduced ferredoxins is replenished afterward. The Complex I-IV of electron transfer phosphorylation and V/A-type H^+/Na^+ transporting ATPase are found in all MAGs, responsible for chemiosmotic energy-conservation, transferring electrons of NADHs generated from central carbon metabolism to terminal electron acceptors and producing ATPs[7].

Fe uptake

From the community analysis, the major microbial players for Fe storage and biosynthesis in the microbial cells are Bacteroidetes (for Fe^{2+} and siderophore uptake), Alphaproteobacteria (for siderophore uptake), Betaproteobacteria (for Fe^{3+} uptake), Deltaproteobacteria (for Fe^{3+} uptake), and Gammaproteobacteria (for Fe^{3+} and siderophore uptake) (Fig. 3). The uptaken Fe could be stored into the microbial cell and complexed into various intracellular Fe-organic compounds which are of great importance to the cell activities[8]. After the cell death, these organic matters could be released and recycled within the microbial community, which makes the Fe-pool stable for the living of microbial cells.

References:

1 Xu, W., Li, M., Ding, J.-F., Gu, J.-D. & Luo, Z.-H. Bacteria dominate the ammonia-oxidizing community in a hydrothermal vent site at the Mid-Atlantic Ridge of the South Atlantic Ocean. *Appl. Microbiol. Biotechnol.* **98**, 7993-8004 (2014).

2 Caporaso, J. G. *et al.* QIIME allows analysis of high-throughput community sequencing data. *Nat. Methods* **7**, 335-336, doi:10.1038/nmeth.f.303 (2010).

3 Hall, T. BioEdit: an important software for molecular biology. *GERF Bull. Biosci.* **2**, 60-61 (2011).

4 Yarza, P. *et al.* Uniting the classification of cultured and uncultured bacteria and archaea using 16S rRNA gene sequences. *Nat. Rev. Microbiol.* **12**, 635-645, doi:10.1038/nrmicro3330 (2014).

5 Hedderich, R. Energy-Converting [NiFe] Hydrogenases from Archaea and Extremophiles: Ancestors of Complex I. *J. Bioenerg. Biomembr.* **36**, 65-75, doi:10.1023/b:jobb.0000019599.43969.33 (2004).

6 Buckel, W. & Thauer, R. K. Energy conservation via electron bifurcating ferredoxin reduction and proton/Na^+ translocating ferredoxin oxidation. *Biochim. Biophys. Acta* **1827**, 94-113, doi:10.1016/j.bbabio.2012.07.002 (2013).

7 Madigan, M. T., John M. Martinko, Kelly S. Bender, Daniel H. Buckley, and David Allan Stahl. *Brock Biology of Microorganisms*. Fourteenth edition edn, (Pearson, 2015).

8 Li, M. *et al.* Microbial iron uptake as a mechanism for dispersing iron from deep-sea hydrothermal vents. *Nat. Commun.* **5**, 3192, doi:10.1038/ncomms4192 (2014).
