# Supplementary material for: Genome- and Community-Level Interaction Insights into Carbon Utilization and Element Cycling Functions of Hydrothermarchaeota in Hydrothermal Sediment
Source: mSystems. 2020 Jan 7;5(1):e00795-19. doi: 10.1128/mSystems.00795-19 (PMC6946796; doi:10.1128/mSystems.00795-19)
Supplement: FIG S1 [file mSystems.00795-19-sf001.pdf]

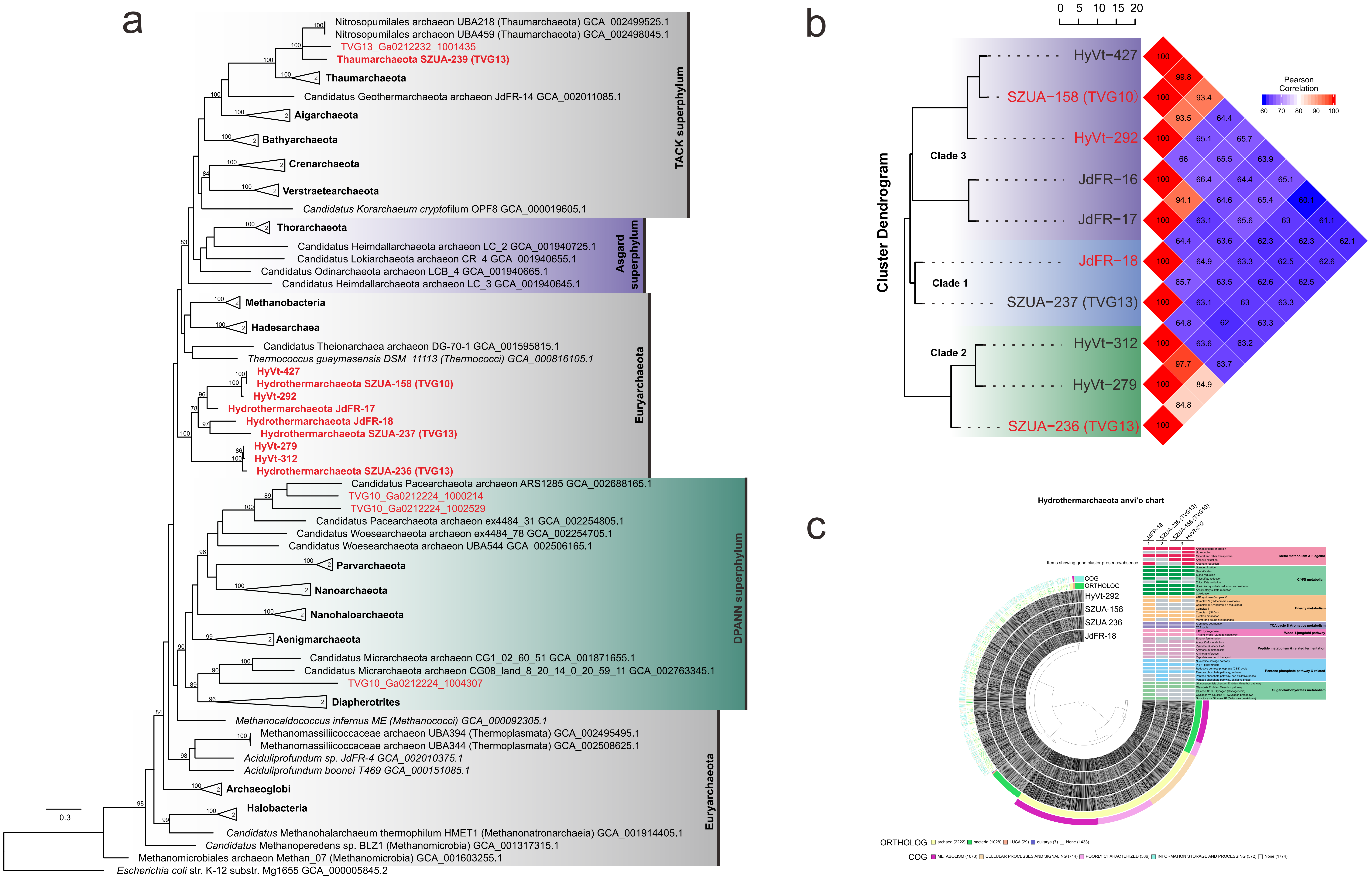

Supplementary Figure S1. The genome taxonomy and genome function of Hydrothermarchaeta. a. Phylogenetic tree of assembled archaeal MAGs and scaffolds based on 12 concatenated ribosomal proteins (L2, L3, L4, L5, L14, L16, L18, L22 and S3, S8, S17, S19 subunits). MAGs and scaffolds with less than 3 ribosomal proteins were pre-excluded. RAxML HPC v.8 was applied to reconstruct phylogeny with the best model as PROTGAMMAILG (suggested by ProtTest 3) and 100 times bootstrap iteration with autoMRE criterion. Escherichia coli K12 was used as the outgroup. Bootstrap supporting values over than 75% were labeled. **b.** Pairwise orthoANI values among Hydrothermarchaeota MAGs. The matrix was reordered according to hierarchical clustering of the orthoANI values. Cluster dendrogram was generated from the matrix of orthoANI values. Red labeled MAGs are of high completeness (> 80%). **c.** Comparative genomics of four Hydrothermarchaeota MAGs. The inner tree topology was assigned according to the matrix of COG functional categories and orthologous groups annotated by eggNOG-mapper. The items showed the presence/absence of gene clusters. The layers showed the presence/absence of specific functions (It is assigned according to the annotation results depicted in Figure 2. If there is one gene found in one specific function, we assign presence to this function).
